# Analysis of mobility homophily in Stockholm based on social network data

**DOI:** 10.1371/journal.pone.0247996

**Published:** 2021-03-09

**Authors:** Cate Heine, Cristina Marquez, Paolo Santi, Marcus Sundberg, Miriam Nordfors, Carlo Ratti

**Affiliations:** 1 Senseable City Lab, Massachusetts Institute of Technology, Cambridge, MA, United States of America; 2 Universidad Carlos III de Madrid, Leganés, Spain; 3 Instituto di Informatica e Telematica del CNR, Pisa, Italy; 4 Department of Urban Planning and Environment, Division of Systems Analysis and Economics, KTH Royal Institute of Technology, Stockholm, Sweden; 5 Strategi- och utvecklingsenheten, Stockholms Stad, Stockholm, Sweden; Peking University Shenzhen Graduate School, CHINA

## Abstract

We present a novel metric for measuring relative connection between parts of a city using geotagged Twitter data as a proxy for co-occurrence of city residents. We find that socioeconomic similarity is a significant predictor of this connectivity metric, which we call “linkage strength”: neighborhoods that are similar to one another in terms of residents’ median income, education level, and (to a lesser extent) immigration history are more strongly connected in terms of the of people who spend time there, indicating some level of homophily in the way that individuals choose to move throughout a city’s districts.

## Introduction

Cities are defined by the flow of people through them—the boundaries of metropolitan areas are drawn based on commuting patterns and on socioeconomic integration [1]; as people move through cities to work, shop, go to school, and socialize, they spread resources and ideas, tying the city together as a single organism. Some even credit the circulation of people throughout a city with the superlinear production of income and innovation [2]. However, a growing body of literature suggests that this circulation of people may be quite irregular: researchers have used high-granularity geospatial mobility datasets from Twitter or mobile phone data to identify individuals’ trajectories around cities and found that people are significantly more likely to colocate with others “like” them across various socioeconomic dimensions [3–5].

While these person-based studies are valuable in analyzing social structure and identifying lack of interaction between, for example, different ethnic groups [6], there is also value in a more place-based approach. Most existing literature has taken the person-location network created as people move through urban spaces (where individuals and locations are two types of nodes and visits from a person to a location are edges) and projected it into an individual-based network, with links between individuals who co-locate [3–6]; however, we can alternatively project the same network to a person-based network, with links between locations which share frequent visitors [7–10]. By studying the overall connectivity between physical locations in a city, as opposed to co-location between individuals, we can identify weaknesses in the circulation of people throughout a city and study the connectedness of the city as a whole: do people flow evenly through urban areas, connecting all neighborhoods to one another, or are there irregularities by which people connect some parts of a city more closely than others?

Further, studying location-connectivity as opposed to person-connectivity bypasses some of the obstacles that come with more traditional approaches. One such obstacle is that existing literature on segregation in activity spaces either relies on travel diaries and surveys [11, 12], or on dense geospatial datasets that come from social media or mobile phones like the Twitter dataset that we use [3–5, 7, 8]. Survey-based methods produce rich data but are limited by sample size constraints and the reliability of respondents. On the other hand, most mobile phone or social media datasets that track human movement are anonymized; attaching individuals in these datasets to the types of socioeconomic characteristics necessary in order to study homophily of any kind requires rough estimation and/or potentially invasive home location estimation, which in turn requires very dense datasets. Our location-based approach does not rely on home estimation at all; instead, our approach focuses on the socioeconomic and demographic features of neighborhoods or statistical areas (which are often measured and released directly by country-level census organizations) and views people as connectors between those places. Our methodology thus avoids both potential error that comes from estimating socioeconomic attributes via home estimation and potential privacy issues that may come from home location estimation. It also allows us to work with a dataset that is too sparse to perform home location estimation or to identify origin-destination pairs, setting it apart from existing location-based social connectivity literature ([7, 8]) and opening up avenues for this type of research in areas where extremely dense geospatial mobility datasets are not available.

We propose a methodology to study connectivity between neighborhoods in Stockholm, Sweden, specifically as it relates to socioeconomic similarity: does the homophily in travel patterns identified in existing literature cause neighborhoods which are socioeconomically similar to one another to be more connected by the flow of people? First, we present a metric for calculating connectivity, which we call “linkage strength”, between any two neighborhoods in the city using data with high spatial but low temporal resolution. Our metric defines connectivity between two areas by co-occurrence of people in those areas—co-occurrence has been used to measure connectivity between physical places in existing studies in other contexts [13, 14]. Our metric is validated by a strong correlation with commonly used connectivity metrics which use origin/destination (OD) pairs in temporally dense data sets [15, 16]. Second, we use generalized linear regression to study the determinants of this connection between places and their relative importance. We focus on socioeconomic similarity as our variable of interest and control for aspects of Stockholm’s geography and urban structure that induce travel patterns in order to isolate the component of the relationship between socioeconomic similarity and linkage strength that comes from preference. These controls include population distribution, structure of transit networks, and a nuanced accessibility measure. The accessibility measure specifically, which was designed by a research team at KTH [17, 18] in order to account for time and mandatory activity constraints in the way that people move around cities, has never before been used in this way. The results of our analysis are relevant to disciplines beyond sociology; for instance, our results could be applied to the dynamic configuration and orchestration of network slices [19], urban development and planning [20], or transportation [21].

The paper is organized as follows. We introduce the methodology and datasets of our study in the Materials and Methods section. The outcome of the three regression analyses we perform are in the Results section. Finally, conclusions and future research directions are outlined in the Conclusions section.

## Materials and methods

### Data

**Fig 1:**
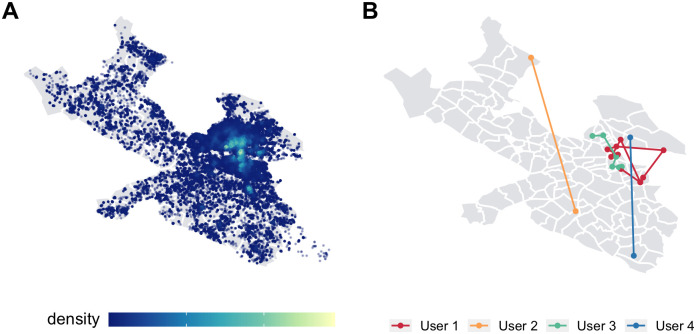
Twitter data. (A) Density of tweets across Stockholm. (B) Trajectories of the geotagged tweets of four random users. User one’s geotagged tweets (red) span eight different stadsdelar across the city, while user four’s tweets (blue) are confined to just two.

Our analysis requires two datasets: one describing movement of people between neighborhoods in Stockholm, and one describing socioeconomic dissimilarity between neighborhoods in Stockholm. In order to understand human mobility across the city, we analyze a set of geotagged tweets in the municipality of Stockholm between January 1, 2016 and April 30, 2019. By looking at successive geotagged tweets, we can understand users’ general mobility spaces—Fig 1 shows the density of Twitter activity across Stockholm as well as the tweet trajectories of four randomly selected users. After filtering out bots, businesses, and other types of uninformative tweets (see S1 Appendix), we analyze 281,863 tweets from 14,478 users.

In order to understand the distribution of socioeconomic characteristics across Stockholm, we look at 2017 census data recorded at the level of Stockholm’s 132 “stadsdelar” (singular: stadsdel)—geographical units containing an average of around 6,000 residents. By aggregating tweet locations up to the stadsdel level, we are able to compare movement across neighborhoods to the socioeconomic characteristics of those neighborhoods; namely, we compare a connectivity metric calculated from Twitter data which we call “linkage strength” (described below) with dissimilarity between pairs of stadsdelar in three socio-economic features: income per person, the percentage of their population that was born in another country, and the percentage of their population that attended some amount of post secondary school (source: City of Stockholm municipality’s Statistical Information Service).

### Calculating linkage strength

In this study, we define connection, or linkage strength, between two areas in terms of shared Twitter users. Users who tweet often in region A and in region B indicate some social or economic connection between the two regions (even if it occurs over the span of weeks) and some colocation of their residents. In order to quantify this type of connection between two locations *A* and *B*, we use the following formula: Let *U*_*A*_ be the set of users who tweet at least once in location *A*. Let *x*_*iA*_ be the number of times user *i* tweets in location *A*. We define *f*_*A*,*B*_ to be the connection, or linkage strength, between location *A* and location *B* created by Twitter users as follows:

$$\begin{array}{c} f_{A,B}=\sum_{i\in U_{A}}\min\left\{x_{i,A},x_{i,B}\right\}. \end{array}$$ (1)

**Fig 2:**
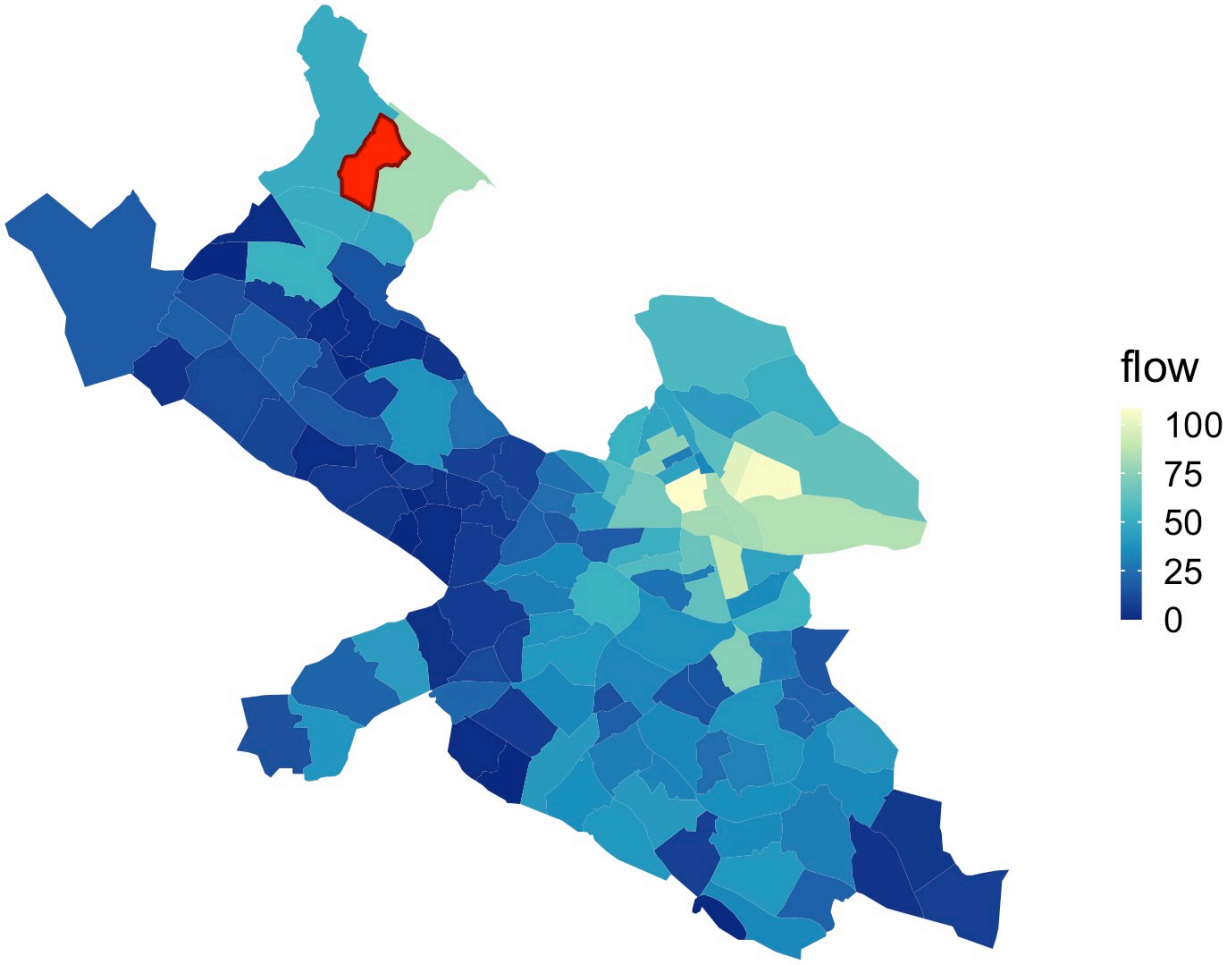
Linkage strength between example stadsdel and all other stadsdelar. Color of each stadsdel represents the value of its linkage strength with the stadsdel highlighted in red, where lighter stadsdelar are more strongly connected to the stadsdel in red and darker stadsdelar are more weakly connected. Note that there is strong connection between the example stadsdel and the stadsdelar in the city center of Stockholm—as these central stadsdelar have high levels of Twitter activity in general, they have strong connection to all other stadsdelar—we control for this difference in Twitter activity volume in our model.

In other words, if a user tweets *x*_*i*,*A*_ times in location *A* and *x*_*i*,*B*_ times in location *B*, they add min{*x*_*i*,*A*_, *x*_*i*,*B*_} units of linkage strength between *A* and *B*. Informally, users who spend time in both locations can be thought of as spreading resources or ideas between the two areas or forming personal connections between residents of the two areas. We take the minimum of *x*_*i*,*A*_ and *x*_*i*,*B*_ because it serves as a bound on the interaction created between the two neighborhoods. See Fig 2 for linkage strength values between an example stadsdel and all other stadsdelar.

#### Relationship with more traditional connectivity metrics

**Fig 3:**
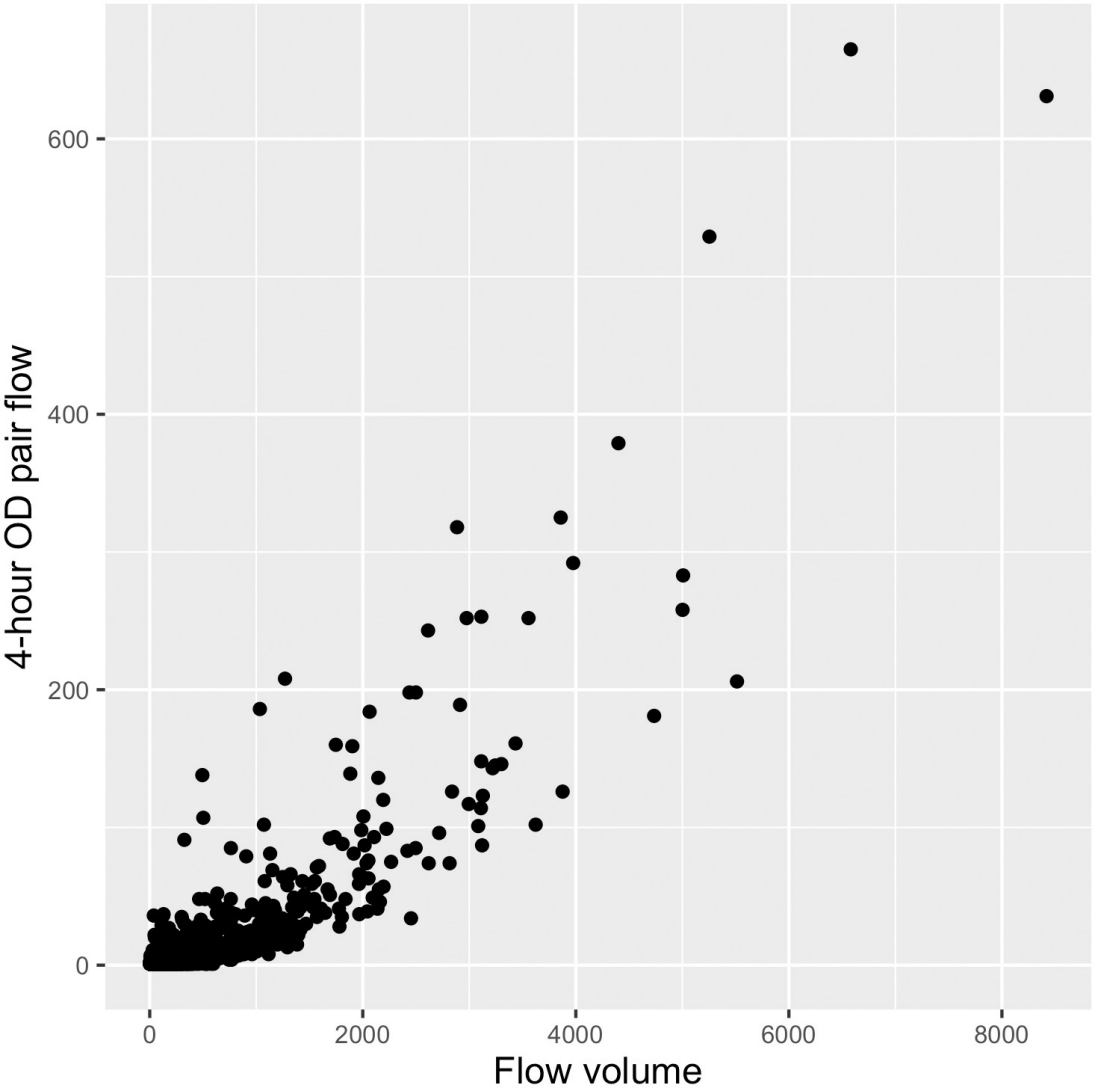
Relationship between linkage strength and existing metric. Our linkage strength metric is on the x-axis, flow as measured by origin-destination pairs from which the same user tweeted within four hours on the y-axis. Pearson correlation coefficient is .853, p-value << .001.

We compare our linkage strength metric to that used in [15, 16], which calculates origin-destination pairs from Twitter data in order to identify commuting patterns and validate travel demand models, respectively. Their metric identifies instances of successive tweets by the same user in two different regions within four hours of one another as an origin-destination trip; flow between two areas is the total number of origin-destination trips between them. They find that the metric performs well in approximating commuting patterns as identified by census survey data and estimated by the SCAG travel demand model. This type of method is infeasible with our dataset, as successive tweets may occur weeks or months apart and do not necessarily correspond to origin-destination pairs; however, we find that our metric is strongly correlated with the metric used in [15, 16], (correlation coefficient .85, p-value << 0.001, see Fig 3) while still allowing us to retain the use all of our data—our metric incorporates information from all roughly 280,000 of our tweets, while the origin-destination flow metric would only be able to incorporate information from 65,521 of those tweets (24,491 OD-pairs).

#### Privacy concerns

Usernames and text associated with the tweets are dropped from our dataset. User ids are hashed to new, random values in order to fully anonymize our data. Further, once linkage strength is calculated, tweets are no longer associated with individuals at all, and trajectories are untraceable in the data. Thus, we remove all identifying information from our data, and we do not add identifying data via home location estimation.

### Model

We use a negative binomial regression to estimate the relationship between our explanatory variables (described below) and linkage strength between pairs of stadsdelar. Linkage strength is a count variable, but it is overdispersed with respect to a Poisson distribution (mean = 102.6, variance = 80056.90); negative binomial regression is appropriate for this kind of overdispersed count data [22]. Because all of our explanatory variables are determined by the origin stadsdel, the destination stadsdel, or a function of the two, we use clustered standard errors as recommended in [23], where the data is clustered on both *A* and *B*—see Discussion for more details. The geospatial nature of our data suggests there could be potential issues with residual spatial autocorrelation [24]; however, we find no residual spatial autocorrelation in any of our three models (see S2 Appendix).

In order to understand differences between stadsdelar in the impact of socioeconomic difference on movement patterns, we also estimate individual negative binomial regression models for each stadsdel. We use fewer explanatory variables—the variables related to origin stadsdel (A) are no longer relevant, as we are looking at only pairs with the same origin stadsdel, and we remove several highly collinear variables, as they contain redundant information and we have fewer degrees of freedom in these smaller models. See Table 1 for the full list of explanatory variables used in the individual-stadsel and all-stadsdelar models. We use the GLM module in the statsmodels python package in order to estimate all of our negaitve binomial regression models.

**Table 1:** Explanatory variables used in individual-stadsdel and all-stadsdel models.

Individual stadsdel models	All stadsdelar models	Variable
✔	✔	Socioeconomic difference
	✔	Transit time
✔	✔	Driving time
	✔	Labor accessibility A
✔	✔	Labor accessibility B
	✔	Log points of interest A
✔	✔	Log points of interest B
	✔	Population A
	✔	Population B
✔	✔	Rank-distance model
	✔	Income A
	✔	Income B
	✔	Education A
	✔	Education B
	✔	Foreign background A
	✔	Foreign background B
	✔	Total linkage strength from A
✔	✔	Total linkage strength from B

### Explanatory variables

#### Variable of interest: Socioeconomic dissimilarity

**Fig 4:**
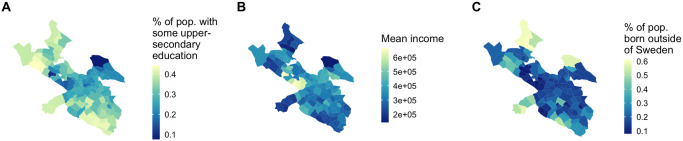
Socioeconomic variables of interest. (A) Percent of the population with some level of post-secondary education. (B) Mean income. (C) Percent of the population first-generation.

We examine whether absolute difference in socioeconomic characteristics between two areas affects the linkage strength between them—are neighborhoods more connected by flow of people to other neighborhoods “like” theirs, in terms of mean income, education levels, and immigrant makeup? We choose three socioeconomic variables for each stadsdel: mean income, percentage of the population with some post secondary education, and percentage of the population classified as “first generation” (those born outside of Sweden). These three characteristics are correlated with one another but distinct—see Fig 4. For each of the three socioeconomic attributes, we run a negative binomial regression on all pairs of stadsdelar. The dependent variable is linkage strength between the two stadsdelar and the variable of interest is absolute difference in their socioeconomic attributes.

#### Control variables

We look at five control variables that can help to account for travel constraints and layout of the city: travel time between two locations, physical accessibility of each location in the city structure, number of points of interest in each location, population of each location, and expected linkage strength in a rank-distance null model of mobility.

*Travel times*. We use the Google Maps API to calculate travel times via driving and public transit between two areas. As longer travel times indicate that areas are more expensive to travel between in terms of both time and money, we expect an inverse relationship between the travel time between two places and linkage strength between them. Analysis of this travel time data indicates heterogeneities in the strength of the transportation network between different neighborhoods—see S3 Appendix for more details.
*Accessibility*. We use a labor accessibility index based on the SCAPER travel demand model [17, 18]. The index acts as a proxy for the proportion of the city’s population that can access the area during the day subject to constraints imposed by daily schedules, mandatory travel locations (work, school) and travel times, which incorporate road and public transit networks as well as traffic information; thus, we expect a positive relationship between accessibility of an area and linkage strength to that area. See S4 Appendix for more details. The index is reported at the EMME node level, which we aggregate up to the stadsdel level by assigning each stadsdel the average of the accessibility indices attributed to the EMME nodes inside of it.
*Points of Interest (POIs)*. We download information on points of interest in each stadsdel from OpenStreetMap. Points of interests include shops, restaurants, tourism sites, parks, among other types of places. As points of interest draw visitors to an area, they could help to explain movement around Stockholm. In order to specifically capture points of interest that would indicate attractiveness of an area, we filter out passive POIs which do not serve as a draw to an area, such as trash cans and surveillance cameras—see S1 Appendix for full list of POI categories. Existing work has shown that OSM data is highly positionally accurate but incomplete (for example [25], found that only 70% of US schools identified in an authoritative national dataset were mapped in OSM); we assume that level of incompleteness is uniform across Stockholm [26].
*Population*. We use 2017 population counts from the Swedish census (source: City of Stockholm municipality’s Statistical Information Service).
*Rank-distance null model*. We estimate expected travel linkage strengths between areas of Stockholm using the rank-distance model in [27]. This models movement between two places *A* and *B* as inversely proportional to rank_*A*_(*B*), where rank_*A*_(*B*) is the number of points of interest closer to *A* than *B* is to *A*. In [27], Noulas et al. find the rank-distance model to fit better than other commonly-used models of human movement, such as gravity models, for within-city travel patterns. Estimating expected linkage strength using this model allows us to account for movement between two places that have only to do with the distribution of amenities across Stockholm, helping us to further isolate choice in the relationship between socioeconomic similarity and linkage strength.

We also include total Twitter activity and the individual socioeconomic characteristics of each stadsdel. In summary, the explanatory variables for the individual-stadsdel and all-stadsdel models are reported in Table 1. There do exist some correlations between our dependent variables, but they do not result in severe multicollinearity issues—see S2 Appendix for full details.

Since our measure of linkage strength is symmetric, the characteristics of each location should contribute equally to it; for this reason, we fix the coefficients on socioeconomic characteristics, accessibility, points of interest, population, total amount of linkage strength and total number of tweets to be the same for both locations (e.g., the coefficient on population of stadsdel A is equal to the coefficient on population of stadsdel B) by including their sums instead of their individual values. We also perform a log transform on accessibility, points of interest, population, and total number of tweets in order to capture the empirical relationship between those variables and our linkage strength metric.

## Results

The outcome of our regression analysis is detailed in Table 2. Controlling for covariates, we see that education and income have significant effects on the linkage strength between two regions at the level *α* = .001, while foreign background has a signficant effect on linkage strength at *α* = .1. For every standard deviation increase in socioeconomic difference, we see total linkage strength multiplied by the exponential of the given coefficient; thus, for every standard deviation increase in income difference between A and B (85,525.27 Swedish krona, equivalent to around 10,251 USD), linkage strength between A and B is multiplied by *e*^−0.097^ = .91, leading to a 8.9% decrease in linkage strength between A and B. For every standard deviation increase in post-secondary education difference between A and B, we see a 10.0% decrease in linkage strength between A and B. For every standard deviation increase in immigrant makeup difference between A and B, we see a 3.4% decrease in linkage strength between A and B. Socioeconomic differences between neighborhoods thus produce statistically significant barriers to linkage strength throughout the city—in the case of income and education, quite stark barriers; in the case of immigration status, relatively weaker barriers.

**Table 2:** All-stadsdel model results.

	*linkage strength between A and B*		
	Income	Education	Foreign background
Constant	3.528*** (0.040)	3.528*** (0.040)	3.530*** (0.040)
Income difference	-0.093*** (0.027)		
Education difference		-0.105*** (0.021)	
Foreign background difference			-0.035* (0.020)
Transit time	-0.144*** (0.031)	-0.138*** (0.030)	-0.140*** (0.031)
Driving time	-0.157*** (0.023)	-0.147*** (0.023)	-0.163*** (0.023)
Log labor accessibility A, log labor accessibility B	0.225*** (0.054)	0.235*** (0.056)	0.232*** (0.056)
Log points of interest A, log points of interest B	0.223*** (0.072)	0.223*** (0.071)	0.238*** (0.070)
Log population A, log population B	0.033 (0.031)	0.030 (0.031)	0.024 (0.031)
Rank-distance model	0.073*** (0.041)	0.071*** (0.014)	0.075*** (0.014)
Income A, income B	-0.075* (0.043)	-0.126*** (0.040)	-0.137*** (0.042)
Education A, education B	-0.077** (0.032)	-0.088*** (0.032)	-0.089*** (0.032)
Foreign background A,	0.114*** (0.039)	0.114*** (0.035)	0.076** (0.038)
Total Twitter activity in A, total Twitter activity in B	0.939*** (0.070)	0.937*** (0.070)	0.935***(0.070)

Note:

*p<0.1;

**p<0.05;

***p<0.01

Importantly, driving and transit time are also significant predictors of linkage strength, and have even larger effects than socioeconomic difference—longer travel times by either mode of transportation are associated with significantly less linkage strength. This indicates that each serves a separate and significant role in connecting physical locations in the city. Strengthening physical infrastructure between parts of the city with low linkage strength could similarly serve to strengthen connectivity between dissimilar places: our results suggest that a decrease of one standard deviation in transit time (about fourteen minutes) is associated with about a 13.4% increase in linkage strength between two stadsdelar.

### Individual-stadsdel models

**Fig 5:**
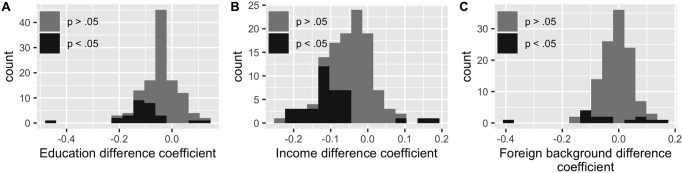
Histograms of individual stadsdel model coefficients. Histogram of coefficients on education (A), income (B), and foreign background (C) difference in individual statsdelar models. In the individual-stadsdel income and education models, more negative coefficients are statistically significant at the *p* < 0.5 level and there are larger negative coefficients than positive ones, which is consistent with the overall trend of a negative relationship between socioeconomic difference and linkage strength. However, this trend is less clear in the foreign background models.

**Fig 6:**
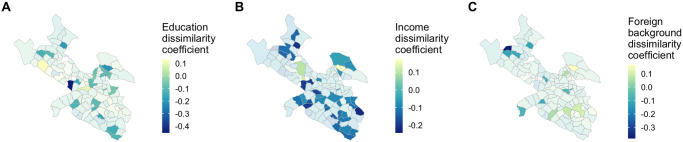
Individual model coefficients. Socioeconomic difference coefficients in the individual-stadsdel education (A), income (B), and foreign background (C) models. Transparent stadsdelar have statistically insignificant coefficients at the .05 level.

We find that predictive effect of socioeconomic difference varies by stadsdel, and is even insignificant in some. In most stadsdelar where socioeconomic difference does have a significant predictive effect on linkage strength, that effect is negative, indicating that neighborhoods are more likely to be connected to other neighborhoods with a similar socioeconomic makeup, consistent with our full-city model. However, there is also a significantly positive effect in some stadsdelar—some neighborhoods are significantly more likely to be connected to neighborhoods which are different from them, contrary to the overall trend in Stockholm (see Fig 5). Fig 6 shows the size of the effect in stadsdelar where it is significant (stadsdelar with no significant effect are shown in gray). As in the all-stadsdel models, the homophily effect is weakest in the foreign background models—education and income seem to have a much stronger effect on connection between stadsdelar.

## Discussion

We have identified significantly stronger linkage between neighborhoods of similar income, education levels, and immigrant makeup. This relationship persists even when controlling for factors induced by the structure and layout of the city, such as transit time between places and intervening opportunities, indicating some level of homophily in the way that individuals choose to move through neighborhoods. This lack of linkage strength between neighborhoods with different socioeconomic characteristics has important implications for social segregation in Stockholm. Researchers have already identified strong residential segregation between ethnic Swedes and immigrants to Sweden and between socioeconomic groups [6]; our results suggest that this segregation persists in activity spaces as well. Further, our results seem to suggest that while difference in immigrant makeup does have a significant effect on connectivity between two stadsdel, it is a small one in comparison to the effects of income and education level. The strong immigrant/non-immigrant residential segregation identified in [6] may weaken as Stockholm residents move through daily activity spaces.

Policymakers have already instituted various policies to try to ameliorate residential divides. For example, the Swedish Migration Agency discourages new immigrants to the city from living in certain socioeconomically challenged neighborhoods by withholding some state benefits if they choose to do so [28]. The neighborhoods in Stockholm specifically that are included in this provision—parts of Rinkeby, Husby, and Tensta—have some of the strongest income, education *and* foreign background dissimilarity coefficients in Stockholm, suggesting that they aren’t well connected to the rest of the city in terms of linkage strength; our results suggest that encouraging new migrants to move elsewhere could potentially have the desired effect of faster integration into Sweden in that they may be more likely to be exposed to people from all across the city [29]. However, our results also suggest that socioeconomically similar communities are connected to one another regardless of physical proximity and accessibility, implying that residential integration alone may not be enough to break down social barriers in Stockholm. Creating public spaces or mixed-income housing units that link areas of different income and education level or immigration background could help to break down existing limits to city connectivity beyond residential segregation.

In our individual-stadsdel models, we have found heterogeneities in the effect of socioeconomic dissimilarity on city connectivity across neighborhoods: consistent with the overall model, most significant socioeconomic difference coefficients in the income and education models were negative, but there were still some stadsdelar with significant, positive coefficients, indicating that they are more likley to be strongly connected to stadsdlar *different* from them by the flow of people. Using the individual-stadsdel models, we were able to identify which stadsdelar have the most significant homophily effect. These stadsdelar could potentially be areas of interest for Stockholm city planners as they plan for activities and spaces that will foster integration.

While the results are promising, it is important that we recognize potential biases in the Twitter data used in this study. Direct demographic information of Twitter users are not available, but language processing studies and formal surveys have found that Twitter’s user population is, in general, younger, more educated, and wealthier than the general population [30, 31]. Further, while geotagged Tweets can serve as a proxy for user location [32], they are not ground-truth: Twitter users opt in to location-sharing, so they may not share the location of every tweet they send and they most likely do not tweet from every location they go to. Selection biases come from both layers of this—the locations that users choose to report may not be representative of their full distribution of tweeting locations, and the locations from which users tweet may not be representative of their full travel trajectories. That being said [33], compares an analysis of six million geotagged tweets across Australia to existing analyses of call data records (which do not contain the previously discussed Twitter biases) to find that Twitter data is, in fact, a useful proxy for human mobility. Their analysis suggests that any biases in our Twitter data may have only limited impact on the results. Future work replicating our findings with, for instance, call detail records from distinct operators [34] could shed more light on this. Finally, Twitter data is known be dominated by certain users, with a small subset of users producing the majority of tweets [30]. In order to ensure that our results are not sensitive to a few, highly influential users, we repeated our analysis with the additional constraint that we filtered out repeated tweets from the same user in a given stadsdel on a given day, ensuring that high-volume users are not over-represented in the data (a process similar to that in [35]). This analysis produced no major changes in the fitted coefficients of our models—see S1 Appendix for full results.

Our distance calculations pose a further limitation to our analysis—travel times were calculated using the geographic centroid of each stadsdel, which may not be representative of travel times between all points in the stadsdelar. Further research could help to illuminate exactly what biases arise from this, if any (see [36]).

Our data is cross-classified and multilevel in structure: each observation *f*_*A*,*B*_ belongs to a cluster of observations associated with stadsdel *A* and a cluster of observations associated with stadsdel *B*. The natural statistical dependencies that will occur in data of this structure are known to cause deflated standard errors and thus over-rejection of the null hypothesis. We choose to account for this using clustered standard errors as recommended in [23], where the data is clustered on both *A* and *B*. While strategies such as multilevel modeling have been shown to be even more effective at reducing over-rejection of the null hypothesis than cluster-adjusted standard errors, we believe that a single level model is sufficient and preferable in our context due to its simplicity and interpretability [37]. It should be noted that our data’s cross-classified structure may cause bias in coefficients on level 2 variables (variables associated with an individual stadsdel); however, we do not attempt to interpret any level 2 variables in this case [38].

It is worth exploring more deeply the causal mechanisms behind the lack of connection between dissimilar places that is demonstrated by our results. Specifically, analyzing the relationship between flow of people between places and infrastructure allowing for that flow (e.g., public transit and road networks) could have important implications for urban design, transportation planning, and the efficient orchestration of network slices. Using place-based measures like ours as opposed to person-based measures is especially amenable to this kind of analysis and planning, as they allow for the identification of weak spots in connectivity of the physical environment, as opposed to other measures of social segregation which look at lack of connectivity in social networks—a metric agnostic of the physical environment.

## Supporting information

S1 Appendix Data pre-processing. We discuss the cleaning process of our Twitter and point of interest data.
(PDF)

S2 Appendix Verification of certain model assumptions. We show that our model does not suffer significantly from bias related to residual spatial autocorrelation or multicollinearity.
(PDF)

S3 Appendix Travel time data. We describe the travel time data used in our model.
(PDF)

S4 Appendix Labor accessibility index. We describe the labor accessibility index used in our model and show how it is distributed across the city of Stockholm.
(PDF)

## References

[1] Bureau USC. Metropolitan and Micropolitan About; 2015. Available from: https://www.census.gov/programs-surveys/metro-micro/about.html.

[2] Bettencourt L. The Origins of Scaling in Cities. Science (New York, NY). 2013;340:1438–1441. 10.1126/science.1235823 23788793

[3] Bora N, Chang YH, Maheswaran R. Mobility patterns and user dynamics in racially segregated geographies of US cities. In: International Conference on Social Computing, Behavioral-Cultural Modeling, and Prediction. Springer; 2014. p. 11–18.

[4] Wang Q, Phillips NE, Small ML, Sampson RJ. Urban mobility and neighborhood isolation in America’s 50 largest cities. Proceedings of the National Academy of Sciences of the United States of America. 2018;115(30):7735–7740. 10.1073/pnas.1802537115 29987019 PMC6065036

[5] Xu Y, Belyi A, Santi P, Ratti C. Quantifying segregation in an integrated urban physical-social space. Journal of the Royal Society Interface. 2019;16(160). 10.1098/rsif.2019.0536 PMC6893495 31744420

[6] Rokem J, Vaughan L. Geographies of ethnic segregation in Stockholm: The role of mobility and co-presence in shaping the ‘diverse’ city. Urban Studies. 2019;56(12):2426–2446. 10.1177/0042098018795561

[7] Prestby T, App J, Kang Y, Gao S. Understanding neighborhood isolation through spatial interaction network analysis using location big data. Environment and Planning A: Economy and Space. 2020;52(6):1027–1031. 10.1177/0308518X19891911

[8] Dorman M, Svoray T, Kloog I. How does socio-economic and demographic dissimilarity determine physical and virtual segregation? Journal of Spatial Information Science. 2020;21(21). 10.5311/JOSIS.2020.21.587

[9] Zhang W, Thill JC. Detecting and visualizing cohesive activity-travel patterns: A network analysis approach. Computers, Environment and Urban Systems. 2017;66:117–129. 10.1016/j.compenvurbsys.2017.08.004

[10] Zhang M, Zhang W, Pang H, et al. Identifying the local and regional travel effects of activity centers in the Austin, Texas area. Southwest Region University Transportation Center (US); 2015.

[11] Browning CR, Calder CA, Krivo LJ, Smith AL, Boettner B. Socioeconomic segregation of activity spaces in urban neighborhoods: Does shared residence mean shared routines? The Russell Sage Foundation Journal of the Social Sciences (RSF). 2017;3(2):210–231. 29034322 10.7758/RSF.2017.3.2.09 PMC5640327

[12] Li F, Wang D. Measuring urban segregation based on individuals’ daily activity patterns: A multidimensional approach. Environment and Planning A. 2017;49(2):467–486. 10.1177/0308518X16673213

[13] Zhang W, Thill JC. Mesoscale Structures in World City Networks. Annals of the American Association of Geographers. 2019;109(3):887–908. 10.1080/24694452.2018.1484684

[14] Longley PA, Adnan M. Geo-temporal Twitter demographics. International Journal of Geographical Information Science. 2016;30(2):369–389. 10.1080/13658816.2015.1089441

[15] Gao S, Yang J, Yan B, Hu Y, Janowicz K, McKenzie G. Detecting Origin-Destination Mobility Flows From Geotagged Tweets in Greater Los Angeles Area. Eighth International Conference on Geographic Information Science (GIScience 2014). 2014; p. 0–4.

[16] Lee JH, Gao S, Goulias K. Can Twitter data be used to validate travel demand models? In: Proceedings of the 14th International Conference on Travel Behaviour Research (ICTBR 2015). Windsor, UK; 2015.

[17] Jonsson D, Karlström A, Oshyani MF, Olsson P. Reconciling User Benefit and Time-Geography-Based Individual Accessibility Measures. Environment and Planning B: Planning and Design. 2014;41(6):1031–1043. 10.1068/b130069p

[18] Västberg OB, Karlström A, Jonsson D, Sundberg M. A dynamic discrete choice activity-based travel demand model. Transportation Science. 2020;54(1):21–41.

[19] Marquez C, Gramaglia M, Fiore M, Banchs A, Ziemlicki C, Smoreda Z. Not All Apps Are Created Equal: Analysis of Spatiotemporal Heterogeneity in Nationwide Mobile Service Usage. In: Proceedings of the 13th International Conference on Emerging Networking EXperiments and Technologies (ACM CoNEXT 2017). Incheon, Republic of Korea; 2017. p. 180–186.

[20] Huang Q, Wong DWS. Activity patterns, socioeconomic status and urban spatial structure: what can social media data tell us? International Journal of Geographical Information Science. 2016;30(9):1873–1898.

[21] van Eggermond MAB, Chen H, Erath A, Cebrian M. Investigating the potential of social network data for transport demand models; 2015. Transportation Research Board 95th Annual Meeting, United States.

[22] Hilbe JM. Negative Binomial Regression. 2nd ed. Cambridge University Press; 2011.

[23] Abadie A, Athey S, Imbens GW, Wooldridge J. When Should You Adjust Standard Errors for Clustering? National Bureau of Economic Research; 2017. 24003.

[24] Fotheringham AS. The Problem of Spatial Autocorrelation and Local Spatial Statistics. Geographical Analysis. 2009;41(4):398–403. 10.1111/j.1538-4632.2009.00767.x

[25] Jackson SP, Mullen W, Agouris P, Crooks A, Croitoru A, Stefanidis A. Assessing completeness and spatial error of features in volunteered geographic information. ISPRS International Journal of Geo-Information. 2013;2(2):507–530. 10.3390/ijgi2020507

[26] El-Ashmawy KLA. Testing the positional accuracy of OpenStreetMap data for mapping applications. Geodesy and Cartography. 2016;42(1):25–30. 10.3846/20296991.2015.1160493

[27] Noulas A, Scellato S, Lambiotte R, Pontil M, Mascolo C. A tale of many cities: Universal patterns in human urban mobility. PLoS ONE. 2012;7(5). 10.1371/journal.pone.0037027 22666339 PMC3362592

[28] Your own accommodation; 2020. Available from: https://www.migrationsverket.se/English/Private-individuals/Protection-and-asylum-in-Sweden/While-you-are-waiting-for-a-decision/Accommodation/Your-own-accommodation.html.

[29] Areas that may affect the right to compensation for asylum seekers; 2020. Available from: https://www.migrationsverket.se/English/Private-individuals/Protection-and-asylum-in-Sweden/While-you-are-waiting-for-a-decision/Accommodation/Your-own-accommodation/Areas-that-may-affect-the-right-to-compensation-for-asylum-seekers.html.

[30] Wojcik S, Hughes A. Sizing Up Twitter Users; 2019. Available from: https://www.pewresearch.org/internet/2019/04/24/sizing-up-twitter-users/.

[31] Longley PA, Adnan M, Lansley G. The geotemporal demographics of twitter usage. Environment and Planning A. 2015;47(2):465–484. 10.1068/a130122p

[32] Hawelka B, Sitko I, Beinat E, Sobolevsky S, Kazakopoulos P, Ratti C. Geo-located Twitter as proxy for global mobility patterns. Cartography and Geographic Information Science. 2014;41(3):260–271. 10.1080/15230406.2014.890072 27019645 PMC4786829

[33] Jurdak R, Zhao K, Liu J, Abou Jaoude M, Cameron M, Newth D. Understanding Human Mobility from Twitter. PLoS ONE. 2015;10. 10.1371/journal.pone.0131469 PMC4496063 26154597

[34] Moyano A, Moya-Gómez B, Gutiérrez J. Access and egress times to high-speed rail stations: a spatiotemporal accessibility analysis. Journal of Transport Geography. 2018;73:84–93. 10.1016/j.jtrangeo.2018.10.010

[35] Gao S, Janowicz K, Montello DR, Hu Y, Yang JA, McKenzie G, et al. A data-synthesis-driven method for detecting and extracting vague cognitive regions. International Journal of Geographical Information Science. 2017;31(6):1245–1271.

[36] Jiang Y, Li Z, Ye X. Understanding demographic and socioeconomic biases of geotagged Twitter users at the county level. Cartography and Geographic Information Science. 2019;46(3):228–242. 10.1080/15230406.2018.1434834

[37] Cheah BC. Clustering standard errors or modeling multilevel data. University of Columbia. 2009; p. 2–4.

[38] Johnson BD. Cross-Classified Multilevel Models: An Application to the Criminal Case Processing of Indicted Terrorists. Journal of Quantitative Criminology. 2012;28(1):163–189. 10.1007/s10940-011-9157-3

