# Supplementary material for: Analysis of mobility homophily in Stockholm based on social network data
Source: PLoS One. 2021 Mar 9;16(3):e0247996. doi: 10.1371/journal.pone.0247996 (PMC7943013; doi:10.1371/journal.pone.0247996)
Supplement: S1 Appendix — We discuss the cleaning process of our Twitter and point of interest data. (PDF) [file pone.0247996.s001.pdf]

1 Twitter data

In order to ensure that we are capturing relevant information from the Twitter dataset, we filter out several types of data. We remove 79,288 tweets that contain invalid or null latitude/longitude coordinates and thus provide no useful information. We also remove 62,891 tweets which utilize the Instagram or Twitter "Stockholm" tag as a geolocation, providing identical coordinates and no meaningful information about their location.
Finally, we remove 9,005 tweets by accounts that we can identify as high-tweeting bots or businesses based on account information or consistent, repetitive tweets on a periodic basis (examples include a police scanner account, which tweets crime incidence from the location of those crimes and thus does not provide information about the movement of an individual, and a bot which tweets the weather at the same time each morning). In all, we remove about 30.9% of our original dataset based on these criteria.

In order to ensure that our results are not heavily influenced by high tweet volumes from a few users, we run our all-stadsdel model again with the additional pre-processing step that repeated observations from the same user in the same stadsdel on the same day are dropped. After this step, our dataset size drops to 65,825 tweets, but our model results are largely unchanged—see Table 1 for full results.

2 Point of interest (POI) data

We remove several point of interest categories which do not represent destinations that would draw people to a stadsdel, including waste baskets and communications towers. The full list of both included and dropped categories is below.

Table 1. All-stadsdel model results additional preprocessing on Twitter dataset.

	linkage strength between A and B		
	Income	Education	Foreign background
Constant	2.559*** (0.037)	2.560*** (0.037)	2.562*** (0.037)
Income difference	-0.100*** (0.025)		
Education difference		-0.104*** (0.020)	
Foreign background difference			-0.047** (0.020)
Transit time	-0.210*** (0.029)	-0.205*** (0.029)	-0.206*** (0.029)
Driving time	-0.129*** (0.018)	-0.120*** (0.019)	-0.135*** (0.019)
Log labor accessibility A, log labor accessibility B	0.171*** (0.045)	0.181*** (0.046)	0.180*** (0.046)
Log points of interest A, log points of interest B	0.173*** (0.055)	0.172*** (0.054)	0.185*** (0.053)
Log population A, log population B	0.015 (0.032)	0.012 (0.032)	0.0079 (0.032)
Rank-distance model	0.083*** (0.016)	0.081*** (0.015)	0.084*** (0.015)
Income A, income B	-0.076* (0.041)	-0.131*** (0.035)	-0.143*** (0.036)
Education A, education B	-0.048 (0.032)	-0.058* (0.033)	-0.057* (0.032)
Foreign background A, foreign background B	0.116*** (0.042)	0.111*** (0.037)	0.085** (0.040)
Total Twitter activity in A, total Twitter activity in B	0.618*** (0.062)	0.618*** (0.061)	0.613*** (0.061)

Note: *p<0.1; **p<0.05; ***p<0.01

included		dropped
supermarket	embassy	toilet
school	veterinary	bench
fast_food	ruins	windmill
bakery	memorial	water_mill
recycling	doctors	tourist_info
hairdresser	bicycle_rental	waste_basket
nursing_home	monument	comms_tower
cafe	cinema	drinking_water
bench	telephone	vending_machine
post_box	garden_centre	camera_surveillance
theatre	greengrocer	water_tower
viewpoint	beverages	
convenience	mall	
playground	bar	
restaurant	travel_agent	
sports_centre	shoe_shop	
tower	hostel	
hotel	police	
fountain	outdoor_shop	
recycling_paper	dog_park	
pharmacy	caravan_site	
kindergarten	mobile_phone_shop	
optician	jeweller	
atm	nightclub	
bank	car_rental	
artwork	department_store	
bicycle_shop	computer_shop	
post_office	dentist	
butcher	car_sharing	
video_shop	shelter	
clothes	vending_parking	
florist	motel	
pub	park	
camp_site	university	
bookshop	toy_shop	
library	gift_shop	
kiosk	community_centre	
museum	hospital	
picnic_site	stationery	
sports_shop	fire_station	
castle	guesthouse	
archaeological	food_court	
doityourself	theme_park	
furniture_shop	arts_centre	
recycling_glass	zoo	
car_dealership	public_building	
attraction	college	
stadium	water_works	
car_wash	courthouse	
laundry	water_well	
beauty_shop	lighthouse	
newsagent	pitch	
swimming_pool	vending_any	

Table 2. Point of interest categories.
