# Supplementary material for: Analysis of mobility homophily in Stockholm based on social network data
Source: PLoS One. 2021 Mar 9;16(3):e0247996. doi: 10.1371/journal.pone.0247996 (PMC7943013; doi:10.1371/journal.pone.0247996)
Supplement: S2 Appendix — We show that our model does not suffer significantly from bias related to residual spatial autocorrelation or multicollinearity. (PDF) [file pone.0247996.s002.pdf]

1 Multicollinearity

Interpretation of our model coefficients requires a lack of multicollinearity—our regression variables must not be highly correlated with one another. In order to measure multicollinearity, we calculate the variance inflation factor (VIF) for each coefficient and each model. Given R_i^2, the R^2 value of the linear regression of variable i on all other variables, $VIF_i = \frac{1}{1-R_i^2}$. A VIF of 5 or greater is often thought to indicate cause for concern with regard to multicollinearity, while a VIF of 10 or greater is often considered indicative of sever multicollinearity. We find that only one of our variables in one model has VIF greater than 5, and our socioeconomic difference coefficients, which are our primary variables of interest, have VIF less than 2.5. While we do have some correlation between our regression variables, these results indicate that multicollinearity is not a severe problem in our model. See Table 1 for full VIF results.

variable	foreign background model	education model	income model
foreign background difference	2.40		
education difference		1.48	
income difference			1.75
foreign background	5.25	3.72	4.08
education	4.08	3.99	4.03
income	3.05	3.07	4.25
total Twitter activity	2.83	2.81	2.81
POIs	4.36	4.34	4.36
accessibility	4.06	4.07	4.09
population	1.73	1.71	1.73
rank-distance null model	1.22	1.21	1.21
transit time	2.16	2.16	2.17
driving time	1.38	1.41	1.39

Table 1. Variance Inflation Factors for all variables in all 3 models.

2 Independence of observations

Our model contains multiple observations for each stadsdel (one for its connection with every other stadsdel, making 117 observations per stadsdel); there will naturally be statistical dependency between observations associated with the same geometry. We account for this with our clustered standard errors. However, the geospatial nature of our data suggests there could also be potential issues with residual spatial autocorrelation [1]—statistical dependencies between observations that are not associated with the same stadsdel but are physically close to one another. We use the Moran's I statistic using the pysal Python package in order to measure spatial autocorrelation of our models' deviance residuals. Values close to 1 indicate perfect clustering of similar data, and values close to -1 indicate perfect dispersal.

Again, because we have multiple observations per geometry, we will naturally have high spatial autocorrelation if we look at all residuals. Thus, we calculate Moran's I for two different sets of the residuals. Consider the residual of the connection between stadsdel i and stadsdel j, $\hat{\epsilon}_{i,j}$:

1. M_{all} = Moran's I$(\{\bar{\epsilon}_i \forall i\})$, where $\bar{\epsilon}_i := \frac{1}{n}\sum_{j=1}^{n}\hat{\epsilon}_{i,j}$ (Take the mean residual across all observations for each stadsdel. Calculate Moran's I statistic for the spatial distribution of these mean residuals.)

2. M_j = Moran's I($\{\bar{\epsilon}_{i,j} \forall i\}$) (For each stadsdel S, take the residuals for the observations of flow between S and every other stadsdel. Calculate the Moran's I statistic for the spatial distribution of these residuals.)

We find that the Moran's I statistics M_{all} and M_j for all three models and all j is statistically 0, indicating that there is no residual spatial autocorrelation in our models. Moran's I statistics and p-values for each all-stadsdel model are in Table 2.

	Income model	Education model	Foreign background model
Moran's I statistic, M_{all}	0.006	0.007	0.008
p-value	.814	0.799	0.781

Table 2. Moran's I values for our three models. The null hypothesis is random dispersal; p-values $> .05$ indicate that we cannot reject the null hypothesis and the assumption of no residual spatial autocorrelation is satisfied.

References

1. Fotheringham AS. The Problem of Spatial Autocorrelation and Local Spatial Statistics. Geographical Analysis. 2009;41(4):398–403.
