# Supplementary material for: Analysis of mobility homophily in Stockholm based on social network data
Source: PLoS One. 2021 Mar 9;16(3):e0247996. doi: 10.1371/journal.pone.0247996 (PMC7943013; doi:10.1371/journal.pone.0247996)
Supplement: S3 Appendix — We describe the travel time data used in our model. (PDF) [file pone.0247996.s003.pdf]

1 Travel time data

Analysis of the travel time data downloaded from the Google Maps API reveals heterogeneities in city connectivity due to the physical road infrastructure and transit network. Figure 1 shows mean driving speed from each stadsdel to all other stadsdelar. Much of this is defined by the physical layout of the neighborhoods: some of the stadsdelar with the longest mean travel time to other stadsdelar are those on the corners of Stockholm, such as Akalla, Skrubba, Vårberg, and Skärholmen. However, there are some geographic irregularities; for example, there is a band of four stadsdelar that are centrally located but less well connected than those around them. This may be attributable to a combination of geography and infrastructure; these four stadsdelar are located along the water, and traveling from them to anywhere in the southeastern part of Stockholm requires first traveling to and crossing one of just two bridges. The most disconnected locations by this travel speed measure are Farstanäset, Hässelby Villastad, Södra Högalid, Flaten and Stora Essingen.

We also identify the district pairs that are the furthest apart by both public transit and driving times. In Figure 2A we highlight the least connected districts by transit: Hässelby Villastad - Sätra, Farstanäset – Grimsta Hässelby Villastad – Farstanäset, Hässelby Villastad – Vårberg, Hässelby Villastad - Orhem. The most disconnected driving areas (Figure 2B) are: Norra Högalid - Farstanäset, Södra Station – Farstanäset, Farstanäset – Grimsta, Södra Högalid – Farstanäset,Farstanäset - Hässelby Strand. Identifying exactly how the nuances in the transit and road networks of Stockholm play into city connectivity is an area for future work.

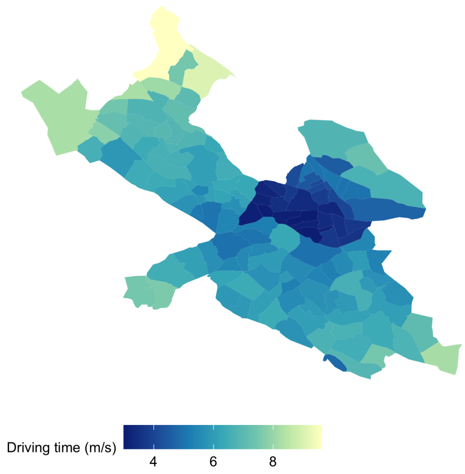

Fig 1. Driving-time connectivity of stadsdelar. Average driving time to the centroid of any other stadsdel.

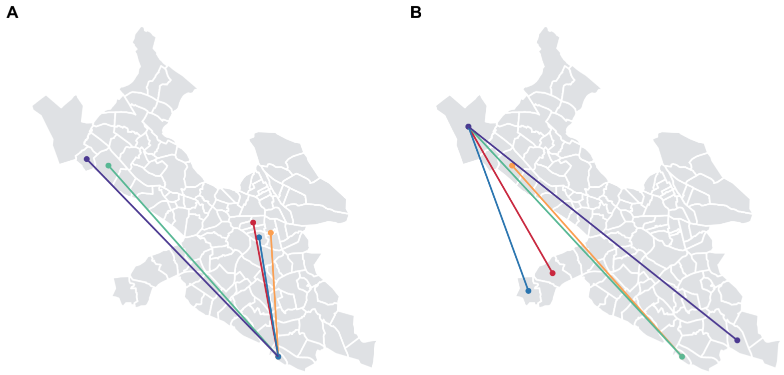

Fig 2. Least connected OD pairs. Pairs of stadsdelar with the highest distance to travel time ratio, based on transit time (A) and driving time (B).
