# Supplementary material for: Analysis of mobility homophily in Stockholm based on social network data
Source: PLoS One. 2021 Mar 9;16(3):e0247996. doi: 10.1371/journal.pone.0247996 (PMC7943013; doi:10.1371/journal.pone.0247996)
Supplement: S4 Appendix — We describe the labor accessibility index used in our model and show how it is distributed across the city of Stockholm. (PDF) [file pone.0247996.s004.pdf]

1 Accessibility index

Accessibility indexes can be used as summary measures to describe the potential to engage in different activities from some place, and typically depend both on the attractiveness of such activities and their spatial configuration, represented through e.g. transport costs or distances. One way of calculating such accessibility measures is through the expected maximum utility derived from a random utility model [?, ?]. The interpretation of the expected maximum utility as an accessibility index has been extended to the realm of dynamic discrete choice models used for activity based travel demand modeling of space-time constrained individuals, leading to recursively defined accessibility measures [?]. Recent advances in the formulation and estimation of such activity based travel demand models have resulted in the SCAPER model [?] which is estimated on travel survey data from Stockholm.

One feature of SCAPER is that it models individuals' activity schedules and corresponding travel demand conditional on home zones and work zones. The model can thus be used to calculate spare-time accessibility measures for individuals conditional on both work and home zone. In the present paper, the estimated model is applied to a typical individual in Stockholm, which is counterfactually assigned to each possible combination of home zone (i) and work zone (j) to calculate conditional spare-time accessibility V_{ij}. The model uses the EMME zonal system, where Stockholm county is represented by 1240 zones with corresponding centroid nodes. While V_{ij} represents an individual's conditional accessibility to spare-time activities, for the pupose of this paper, this measure is aggregated over home locations and the n_i individuals residing there through log-summation to achieve a measure of labor accessibility (V_j) which is conditional on work zone alone

$$V_j = log\left(\sum_i n_i e^{V_{ij}}\right).$$

The calculated labor accessibilities are displayed to the left of Figure 1. This type of aggregation is justifiable if all n_i individuals experience the same accessibility as the typical individual for which the model was applied. We hypothesize that that this

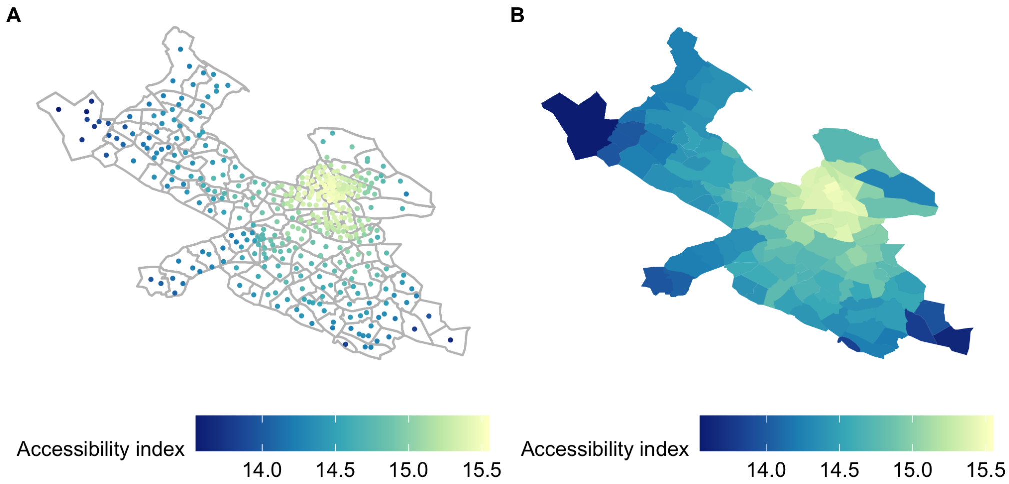

Fig 1. (A) Accessibility index as originally calculated at the EMME node level.(B) Accessibility index aggregated to the stadsdel level; each stadsdel is assigned the mean of the index levels of the nodes it contains.

accessibility variable should constitute a proxy for the total inflow of commuters to zone

j, and furthermore that pairs of regions which have large inflows also have potential for large inter-zonal flows, i.e. we expect a positive relationship between this measure of labor accessibility and flow between zones.
